# Supplementary material for: Simultaneous Induction of Glycolysis and Oxidative Phosphorylation during Activation of Hepatic Stellate Cells Reveals Novel Mitochondrial Targets to Treat Liver Fibrosis
Source: Cells. 2020 Nov 11;9(11):2456. doi: 10.3390/cells9112456 (PMC7697161; doi:10.3390/cells9112456)
Supplement: Supplementary file 1 [file cells-09-02456-s001.pdf]

Tables

Table S1: List of primers and probes used. Gene, probe sequence, forward and reverse primer sequence, and species listed for each of the genes used.

Gene	*Probe Sequence*	*Forward primer*	*Reverse primer*	*Species*
acta2	CTTCACACATAGCTGGAGCAGCTTCTCGA	GCCAGTCGCCATCAGGAAC	CACACCAGAGCTGTGCTGTCTT	Rat
col1a1	TCCTGCTGGTCCCCGAGGAAACA	TGGTGAACGTGGTGTACAAGGT	CAGTATCACCCTTGGCACCAT	Rat
ppar-γ	CCA ACA GCT TCT CCT TCT CGG CCT G	CAC AAT GCC ATC AGG TTT GG	GCT GGT CGA TAT CAC TGG AGA TC	Rat
ppargca1a	CCC CAT TTG AGA ACA AGA CTA TTG AGC GAA CC	GAC CCC AGA GTC ACC AAA TGA	GGC CTG CAG TTC CAG AGA GT	Rat
mtDNA	CGTCGAATACGCCGCAGGACCA	GGAGAATCAGAATTAGTCTCAGGCTTT	GGTGTACTCGGCTATGAAGAATAGG	Rat
β-Actin	CTGGCCGGGACCTGACAGACTACCTC	TGCCCATCTATGAGGGTTACG	CGCTCGGTCAGGATCTTCA	Rat
mfn1	TGCGCACATCCTCCATATATTCTGGCTCT	TCGTTGGGATGCTTCTGCTT	CCAAGAAGTGAAGACATCTTTCCA	Rat
mfn2	TCCCATTGCTCGTCCGGCCA	GGCACATGAAGGTGGCTTTT	CCAAAGCATGGCATTGATCA	Rat
ACTA2	CACTCTTTCTACAATGAGCTTCGTGTTGCCC	GGGACGACATGGAAAAGATCTG	CAGGGTGGGATGCTCTTCA	Human
COL1A1	CCCCAAGGACAAGAGGCATGTCTG	GGCCCAGAAGAACTGGTACATC	CCGCCATACTCGAACTGGAA	Human

Table S2: List of antibodies used. Protein, type of antibody, company and catalog number and dilutions used for western-blot and immunofluorescence of each of the antibodies used.

Protein	*Antibody*	*Company & catalog number*	*Dilution*
PGC1-α	Rabbit polyclonal	Abcam, ab54481	1/1000
VDAC	Rabbit polyclonal	Abcam, ab34726	1/1000
Pex-14	Rabbit polyclonal	Home made, gift	1/1000
α-SMA	Mouse monoclonal	Sigma aldrich, A5228	1/1000
OXPHOS	Mouse monoclonal	Abcam, ab110413	1/500

Supplementary Material

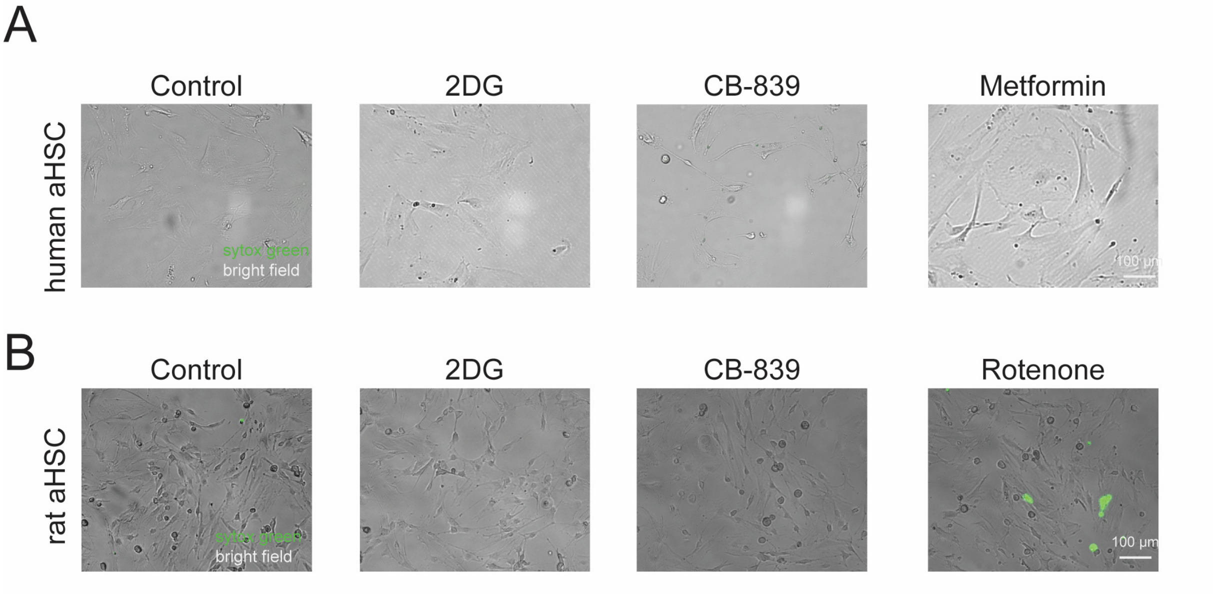

Supplementary Figure S1: Toxicity of inhibitors used in primary human HSC and primary rat HSC. Sytox green staining was used to asses toxicity of glycolysis inhibitor 2DG (2,5 mmol/L) glutaminase inhibitor CB-839 (5 µmol/L), and complex I of ETC inhibitor, rotenone (5 µmol/L) or metformin (2 mmol/L) for A) human aHSC and B) rat aHSC after 72h of treatment. n=3.
